# Cause Analysis and Diagnosis and Treatment of Intestinal Fistulas After Ultrasound-Guided Microwave Ablation of Abdominopelvic Lesions

**DOI:** 10.3389/fsurg.2021.675585

**Published:** 2021-11-19

**Authors:** Shuilian Tan, Xiaoling Yu, Zhigang Cheng, Jing Zhang, Jie Yu, Fangyi Liu, Yuanjin Gao, Runze Linghu, Zhiyu Han, Ping Liang

**Affiliations:** Department of Interventional Ultrasound, Chinese People's Liberation Army General Hospital, Beijing, China

**Keywords:** intestinal fistulas, ultrasound-guided microwave ablation, abdominopelvic, surgical, treatment exploration

## Abstract

**Objective:** To determine the cause and high-risk factors for the development of intestinal fistulas (IFs) after ultrasound-guided microwave ablation (MA) of abdominopelvic lesions, and to identify effective prophylactic and therapeutic actions.

**Methods:** Clinical data were collected from patients with an IF after ultrasound-guided MA of abdominopelvic lesions in our hospital from January 1, 2010 to December 31, 2018. The cause, diagnosis, and treatment of IFs in these patients were analyzed.

**Results:** Among 8,969 patients who underwent ultrasound-guided MA of abdominopelvic lesions, eight patients developed IF after MA, Seven patients were discharged after being cured and one died.

**Conclusion:** Abdominopelvic lesions are close to the intestines, so histories of surgery, radiotherapy, and abdominopelvic infection are high-risk factors for IF development after MA of these lesions. Surgical treatment should be provided as soon as an IF is identified.

## Introduction

Ultrasound-guided microwave ablation (MA) is a minimally invasive method that can result in rapid recovery, short hospital stay, and good tolerance to treatment (1). MA is used widely in the treatment of various solid tumors in abdominal and pelvic cavities, including liver tumors, renal tumors, uterine myomas, adenomyosis, and adrenal-gland tumors (2–6).

Common complications of ultrasound-guided MA of abdominopelvic lesions include hemorrhage, secondary infection at the MA site, diaphragmatic injury, biliary fistulas, pneumothorax, hemopleura, and gastrointestinal injury (7). Intestinal fistulas (IFs) are rare, but relatively serious, complications. Despite their low incidence, IFs are relatively difficult to treat once they develop because further surgery may increase patient trauma and may even affect the survival and prognosis of cancer patients.

We reviewed cases of IFs after MA of abdominopelvic lesions in our hospital and analyzed the primary disease, history and site of IFs, as well as the diagnosis and treatment after IF development in these patients. In this way, we identified the high-risk factors as well as prophylactic and therapeutic measures for IFs.

## Patients and Methods

This was a retrospective study approved by the Ethics Committee of our hospital. All treatments were provided after patients had provided written informed consent.

There were 8,969 patients who underwent ultrasound-guided MA of abdominopelvic lesions in our department from 1 January 2010 to 31 December 2018: 7,240 cases of liver tumor, 464 cases of renal tumor, 60 cases of adrenal-gland tumor, and 1,205 cases of uterine myoma and adenomyosis. Eight patients (three males and five females; 40–84 years) developed an IF. One patient (number 1) had primary liver cancer, 17 years of hepatitis-B infection, 11 years of cirrhosis, and a history of cirrhotic ascites, upper gastrointestinal bleeding (two episodes), and cholecystectomy (due to stones). Also, three patients (numbers 2–4) had renal tumors. Three patients (numbers 5–7) had uterine lesions. One patient (number 8) had metastasis to the right adrenal gland 5 years after resection of a right renal tumor and had received exploratory laparotomy and radiotherapy 1 year before hospital admission. The clinical diagnosis, history, and lesion status (location and dimension) of these eight patients are shown in Table 1. The mean duration of follow-up was 12 months.

**Table 1:** Clinical characteristics of patients with an intestinal fistula.

**Patient no**.	**Sex**	**Age (years)**	**Primary disease**	**Lesion location**	**Lesion size (cm)**	**Medical history**	**History of radiotherapy of the treatment area**
1	M	64	HCC	Liver S8	2.0 × 1.6 1.0 × 1.0	Cholecystectomy	None
2	F	54	Left renal hamartoma	Left kidney (middle region)	4.8 × 4.1	Hysterectomy	None
3	F	84	Left renal clear cell carcinoma	Left kidney (middle and lower regions)	3.3 × 2.9	Radical surgery for ovarian cancer	None
4	M	60	Left lung squamous cell carcinoma	Left kidney (middle region)	5.8 × 5.4 × 4.7	None	None
5	F	40	Adenomyosis	Uterus	Anterior wall, 3.7; posterior wall, 2.2	Cesarean section	None
6	F	41	Adenomyosis	Uterus	Posterior wall, 4.2	Ovarian-teratoma resection	None
7	F	40	Uterine myoma	Uterine posterior wall	5.4 × 5.1 × 4.2	None	None
8	M	62	Right renal clear cell carcinoma	Right adrenal gland	7.6 × 2.6, 7.4 × 2.6	Right-kidney resection	Yes

## MA

MA was undertaken using a KY-2000 system (Canyon Medical, Nanjing, China). This system has a transmission frequency of 2,450 MHz and is equipped with a 15-G water-cooled MA electrode. Different levels of power and MA duration were used based on the diameter and location of the lesion (Table 2). Real-time ultrasound monitoring during MA indicated that the target lesions were covered by strong echoes. MA was terminated if the blood supply was no longer detected in the lesion by contrast-enhanced ultrasound.

**Table 2:** Auxiliary measurements.

**No**	**Ablation site**	**Lesion size (cm)**	**Number of electrodes**	**Power (W) and duration (s)**	**Auxiliary measurements**
1	Liver S8	2.0 × 1.6, 1.0 × 1.0	2	50, 960	Artificial pleural effusion
2	Left kidney (middle region)	4.8 × 4.1	2	40, 660; 45, 120; 50, 180	None
3	Left kidney (middle and lower regions)	3.3 × 2.9	2	50, 390; 45, 600	Temperature measurement
4	Left kidney (middle region)	5.8 × 5.4 × 4.7	2	50, 1440; 60, 1080	Artificial ascites
5	Uterus	Anterior-wall thickness, 3.7; posterior-wall thickness, 2.2	2	50, 920; 30, 160; 40, 100; 60, 180	Intrauterine catheterization
6	Uterus	Posterior-wall thickness, 4.2	2	40, 400; 50, 1870	None
7	Uterine posterior wall	5.4 × 5.1 × 4.2	1	40, 230; 50, 300; 60, 300	None
8	Right adrenal gland	7.6 × 2.6, 7.4 × 2.6	2	50, 1320	Artificial ascites

Auxiliary measurements were applied to five of the eight patients during MA (Table 2). Temperature measurement: the temperature probe was placed at the lateral posterosuperior region of the lesion close to the intestines, and the highest temperature was 48°C. Intrauterine intubation: sterile physiologic (0.9%) saline was injected during MA.

## Results

### Eight Patients IF-Related Symptoms, Examination, Diagnosis, and Treatment

#### Patient Number 1

**Figure 1:**
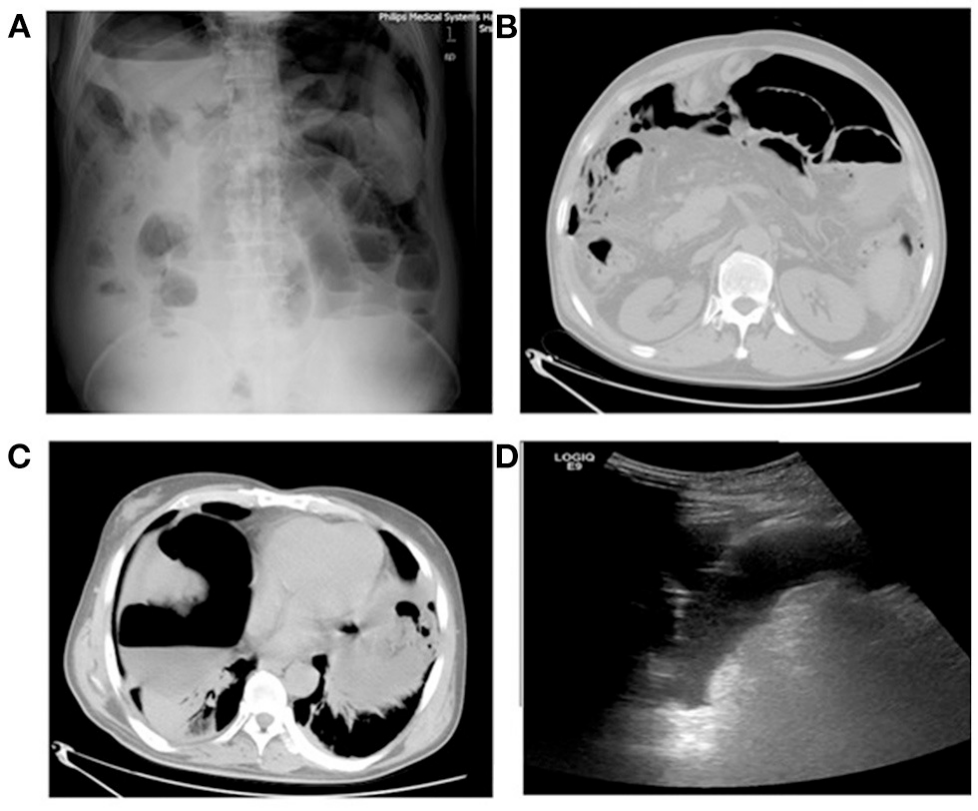
Patient number 1: male, 64 years, hepatocellular carcinoma (S8 segment). Ultrasound examination showed pelvic effusion and poor sound transmission on 4 days after microwave ablation. CT radiography of the abdomen (standing) showed free intraperitoneal air. **(A)** Radiography of the abdomen (standing) 6 days after microwave ablation. **(B)** CT 6 days after microwave ablation. **(C)** CT 6 days after microwave ablation. **(D)** Ultrasound of the abdomen 4 days after microwave ablation.

One day after MA, this liver-cancer patient experienced chills and fever. On day-2, MRI of the abdomen showed complete MA of the lesions without signs of intestinal perforation. On day-3, the patient developed a fever with a maximum temperature of 39.4°C, accompanied by abdominal distension and reduced air release. On day-4, the patient experienced persistent fever and abdominal distension, and ultrasound examination showed a peritoneal effusion (Figure 1D). The patient had peritoneal irritation, abdominal tenderness and rebound tenderness. On day-5, diagnostic abdominal puncture revealed turbid light-yellow liquid. On day-6, abdominal computed tomography (CT) and abdominal radiography in the standing position showed substantial free air in the abdominal cavity (Figures 1A–C). The diagnosis was peritonitis and intestinal perforation.

#### Patients Number 2–4

**Figure 2:**
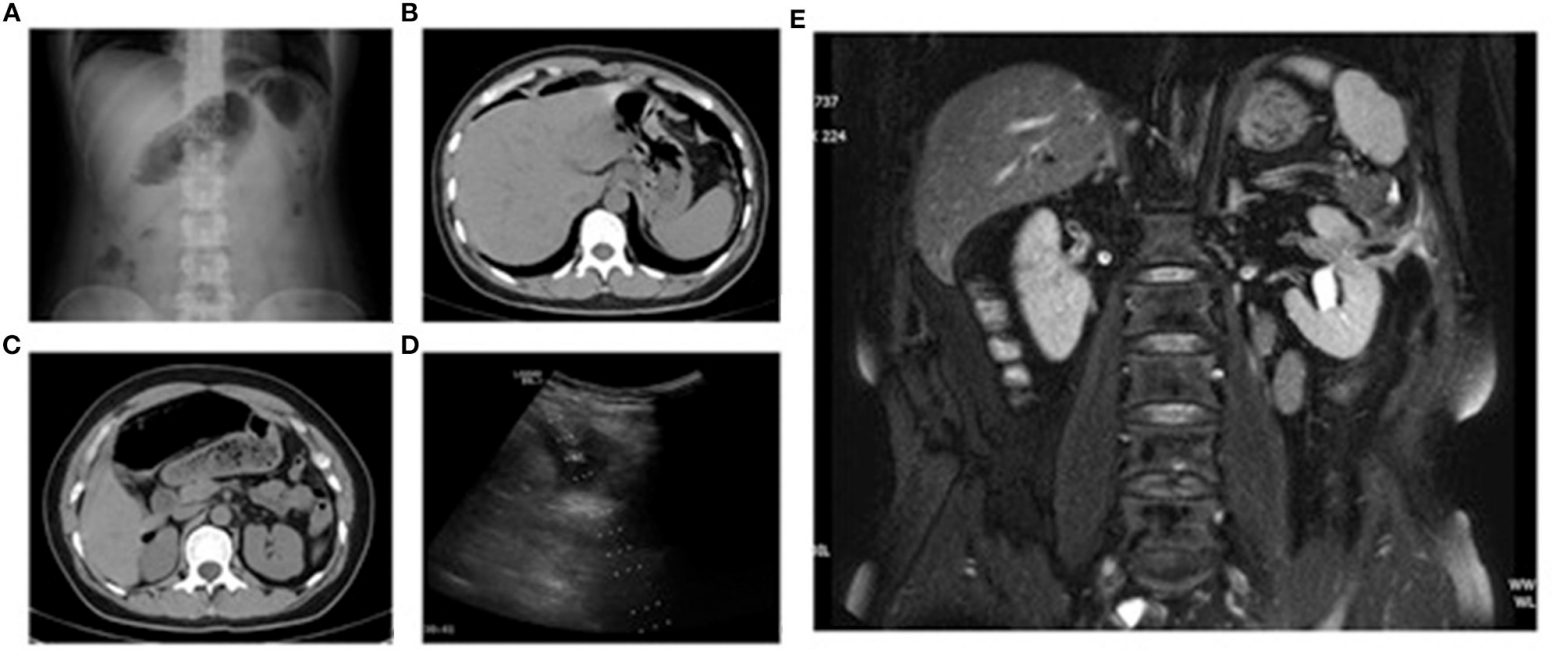
Patient number 7 **(A–D)**: female, 40 years, myoma in the posterior wall of the uterus. Abdominal radiography (standing) and CT 1 day after microwave ablation showed free intraperitoneal air. Ultrasound showed pelvic effusion, and diagnostic paracentesis was carried out. Patient number 4 **(E)**: male, 60 years, left renal metastasis of left-lung squamous cell carcinoma. MRI 52 days after microwave ablation showed left perirenal abscess and fistula between the intestines, ablation area, and urinary system. **(A)** Radiography of the abdomen (standing) 1 day after microwave ablation. **(B)** CT 1 day after microwave ablation. **(C)** CT 1 day after microwave ablation. **(D)** Ultrasound 1 day after microwave ablation. **(E)** MRI 52 days after microwave ablation.

All three patients with a renal tumor experienced abdominal distension 2 days after MA without abdominal pain or peritoneal irritation. Patient number 2 had intermittent, low-grade fever, whereas patient number 4 did not have fever within 1 week after MA. Patient number 2 and 4 had normal white blood cell (WBC) counts. Patient number 3 experienced intermittent post-meridian fever that reached ≤38.6°C. This patient had a WBC count of 13.17 × 100/L and a NE level of 0.865 1 day after MA, but these indices returned to normal after anti-inflammatory treatment. Imaging re-examinations (ultrasound and MRI/CT) of these three patients within 5 days after MA showed complete ablation of lesions without signs of an IF. Patients number 2 and 4 experienced intermittent fever 2 weeks after MA. On day-26 after MA, patient number 2 had a subcutaneous mass of dimension 12 × 8 cm at the needle-insertion site on the left abdominal wall, along with redness, swelling, fever, and pain. Abdominal CT showed an IF with local infection. The diagnosis was a post-MA IF. Patient number 4 experienced fevers on 14 days after MA, but abdominal CT showed no signs of an IF. This patient subsequently had intermittent watery stools (stool mixed with urine) 20 days after MA, as well as intermittent urination and air release 30 days post-MA. Nuclear MRI 52 day's post-MA suggested a left renocolic fistula (Figure 2E). Patient number 3 experienced inguinal discomfort (in the ureteral area) 14 days after MA, along with three intermittent episodes of hematuria, sediment-like necrotic tissues in urine, and intermittent urination with a small amount of air release. Abdominal CT indicated air accumulation in the target lesions of the inferior pole of the left kidney and free air in the renal pelvis. Stools were observed in the urine of patient number 3 on day-20. The patient was diagnosed with a left renocolic fistula.

#### Patients Number 5–7

All three patients with uterine lesions experienced substantial abdominal pain 1 day post-MA, accompanied by peritoneal irritation, abdominal distension, weakened bowel sounds, and fever. Patient number 5 and 6 had normal WBC counts, whereas patient number 7 had a WBC count of 11.79 × 10^9^/L. Ultrasound examination of these three patients revealed pelvic effusion and poor sound transmission (Figure 2D). Ultrasound-guided diagnostic biopsy demonstrated that the effusion was yellow and turbid. Radiography of the abdomen in the standing position and/or CT showed free air in the subdiaphragmatic region and abdominal cavity (Figures 2A–C). These patients were diagnosed with an IF.

#### Patients Number 8

This patient experienced no discomfort after MA of a metastatic tumor in the right adrenal gland and was discharged from hospital. However, the patient experienced intermittent fever 15 days post-MA, and abdominal CT 35 day's post-MA revealed infection of the MA site. Intubation was undertaken to drain intestinal contents. This patient was diagnosed with an IF.

Among these IF patients (Tables 3, 4), seven underwent surgery (Table 5), and six (numbers 2–3 and 5–7) were cured and discharged, whereas one (number 1) died of septic shock 18 days after surgery. One patient (number 4) received conservative treatment (ultrasound-guided puncture and catheter drainage of a left perirenal abscess), and was extubated and discharged after 2 months. An IF developed in the colon of five patients (numbers 1–4 and 8) and in the ileum of three patients (numbers 5–7).

**Table 3:** Symptoms associated with intestinal fistulas.

**No**	**Initial symptom/time of onset (day)**	**Fever/time of onset (day)**	**Peritoneal irritation/time of onset (day)**	**Fecal occult blood /time of onset (day)**	Leukocyte increase (*10^**9**^) /time of onset (day)
1	Chills, 1	1	Yes, 4	Not checked	11.64 and 6
2	Abdominal distension, 2	2	No	Yes, 2	Normal
3	Abdominal distension, 2	2	No	Yes, 8	13.17, 1
4	Abdominal distension, 2	14	No	No	Normal
5	Abdominal pain, 1	1	Yes, 1	Not checked (no stool)	Normal
6	Abdominal pain, 1	1	Yes, 1	Not checked (no stool)	Normal
7	Abdominal pain, 1	1	Yes, 1	Not checked (no stool)	11.79 and 1
8	Fever, 15	15	No	No	Normal

**Table 4:** Imaging and diagnostic data.

**No**	**CT/MRI and time (day)**	**Plain abdominal radiograph (standing)/time (day)**	**Ultrasound/time (day)**	**Diagnostic biopsy/time (day)**	**Time taken for diagnosis (day)**
1	CT, free intraperitoneal air, 6	Free intraperitoneal air, 6	Pelvic effusion, poor sound transmission, 4	Yellow turbid fluid, 5	6
2	CT, needle-path infection, 26	Not checked	Needle-path infection, 26	Not done	26
3	CT, air accumulation in kidney and pelvis, 14	Not checked	No pelvic effusion	Not done	20
4	MRI, left renocolic fistula, 52	Not checked	Perirenal abscess, 53	Yellow turbid fluid, 53	52
5	Not checked	Free intraperitoneal air, 2	Pelvic effusion, 2	Yellow turbid fluid, 2	2
6	CT, subdiaphragmatic free air, 1	Right subdiaphragmatic free air, 1	Pelvic effusion, 1	Yellow turbid fluid, 1	1
7	CT, subdiaphragmatic and intermesenteric free air, 1	Subdiaphragmatic free air, 1	Pelvic effusion, 1	Not done	1
8	CT, abscess and air accumulation at ablation site, 35	Ablation area air–fluid level, 35	Ablation-area abscess, 37	Yellow turbid fluid, 37	37

**Table 5:** Surgical approaches and outcomes.

**No**	**Site of intestinal fistula**	**Surgical approach**	**Outcome**
1	Transverse colon in liver region	Partial resection of transverse colon, end-to-end anastomosis of the ascending colon and transverse colon, jejunostomy	Died
2	Splenic flexure of descending colon	Double-cavity ostomy of the transverse colon on the abdominal wall, and the fistula orifice was repositioned after 4 months	Cured and discharged
3	Descending colon	Left-hemicolon and left-kidney resection, ileostomy, and the fistula orifice was repositioned after 5 months	Cured and discharged
4	Colon	Ultrasound-guided catheter drainage for a left perirenal abscess for 2 months	Cured and discharged
5	Ileum	Partial resection of the ileum, side-to-side anastomosis of the ileum	Cured and discharged
6	Ileum	Partial small-intestine resection, intestinal anastomosis	Cured and discharged
7	Ileum	Partial small-intestine resection, end-to-end anastomosis of the small intestine	Cured and discharged
8	Colon in liver region	Partial resection of the colon, end-to-end anastomosis of the ascending colon and transverse colon	Cured and discharged

## Discussion

Ultrasound-guided MA is minimally invasive and used widely for treatment of various solid tumors in abdominal and pelvic cavities. An IF is a rare (but serious) complication of MA.

Liang et al. reported that the prevalence of an IF after MA of a liver tumor is <0.2% (6). Reports on the prevalence of IF after MA of renal tumor and uterine lesion were mostly case reports (8–15). The prevalence of an IF after MA of an adrenal-gland tumor has not been reported. But IF after MA often requires surgical treatment. An IF is often complicated by peritonitis, abscess, bacteremia, and even sepsis or septic shock, which can be life-threatening.

### High-Risk Factors Analysis for an IF

#### MA Site Is Close to the Intestines

It has been reported that a distance <1 cm between the intestines and MA site for a liver tumor or a distance <5 mm between the intestines and MA for a renal tumor are high-risk factors for intestinal injury (9, 16, 17). If the MA site is close to the intestines, MA poses a direct risk of intestinal damage. Also, if the lesion shrinks after high temperature-induced necrosis, penetration of the microwave electrode tip through the lesion into the adjacent intestines can cause intestinal injury.

#### Misplacement of the Microwave Electrode

Misplacement of the microwave electrode into the intestines during MA can cause intestinal injury due to poor visibility of the electrode tip. This is a result of shielding by the strong echoes generated by air at the MA site.

#### Adhesions

Adhesion resulting from a history of surgery, radiotherapy, abdominal infection, or endometrial displacement is a risk factor for an IF (16, 18). Artificial ascites may fail to separate adhesions. In addition, adhesions can reduce intestinal peristaltic movement, and the intestinal wall is burnt constantly by the heat of the electrode even if it does not penetrate the intestines directly which, eventually, leads to intestinal necrosis and an IF.

#### Previous Radiotherapy

Previous radiotherapy of the proposed MA site and adjacent area results in radiation enteritis and increased intestinal permeability. Post-MA intestinal bacterial translocation causes infection at the MA site and leads to development of a secondary IF.

#### Tumor Invasion

Tumor invasion of the adjacent intestinal tract results in communication between the intestinal lumen and lesions, which results in an IF after MA.

#### Features of the Small Intestine

The small intestine has low air content and the intestinal tract shrinks if compressed. Hence, the small intestine is difficult to visualize by ultrasound (using an abdominal probe), so the microwave electrode can penetrate through the intestinal tract directly and cause injury.

### Diagnosis of an IF

Early diagnosis of IF after MA is important. Early diagnosis and treatment often affect the prognosis of patients. IF is diagnosed primarily based on symptoms, physical signs, imaging, and laboratory tests. The common symptoms and signs of IF are fever, abdominal distension, abdominal pain, peritoneal irritation, peritoneal effusion, and free intraperitoneal air. Patients may exhibit different initial symptoms and signs depending on the location of IF. If a fistula develops in the large intestine, leaking is slow and the leaked contents contain bacteria and air. The initial symptoms of patients are often fever and abdominal distension without obvious peritoneal irritation. Early MRI and ultrasound often reveal no positive results (which was the case for patient number 1). In contrast, if a fistula develops in the small intestine, the intestinal contents contain much fluid and leak fast, and the alkalinity of the leaking intestinal fluid (which contains pancreatic juice, bile, and digestive enzymes) can cause chemical peritonitis. As a result, abdominal pain is often the initial symptom of patients with a fistula in the small intestine, and often the visual analog scale score is >5. Analgesic drugs are unsatisfactory for relieving the pain. Patients with fistulas in the small intestine often experience peritoneal irritation, manifesting as abdominal muscle tension, obvious abdominal tenderness and rebound pain, and weakened/disappearance of bowel sounds, as revealed by physical examination. Increased leakage and secondary infection may result in bacterial peritonitis (patients numbers 5–7). However, the symptoms of peritonitis may be mild or absent in such cases because the intestinal contents do not pass directly through the abdominal cavity as a result of the adhesion.

Further examinations (abdominal radiography in the standing position, CT, ultrasound and/or MRI) should be done if intestinal injury is suspected. An IF often manifests as free air in abdominal and pelvic cavities on abdominal and pelvic CT and abdominal radiography, and the site of intestinal injury may be visible on abdominal and pelvic CT in some cases. Conversely, an IF manifests primarily as free fluid in the abdominopelvic cavity by ultrasound, and the effusion has relatively poor sound transmission. However, further diagnoses may be made after a diagnostic puncture. If leakage of intestinal contents is localized and they do not enter the abdominopelvic cavity directly after IF development, peritoneal irritation is often absent and this condition is more common in an injury to the descending colon. The initial symptom is often non-specific abdominal distension, which occurs <3 days after MA but disappears after 3 days. The symptom of fever is often delayed, which occurs if there is leakage of a relatively large amount of intestinal contents after MA and is mostly evident 10 days after MA. The symptom of fever gradually exacerbates over time but is often not accompanied by chills. Due to the application of antibiotics, the WBC count and neutrophil count are often normal or slightly higher than normal. Blood-culture results can be positive with increasing leakage and development of bacteremia due to local secondary infection. There are no important specific manifestations by imaging <1 week after MA. Infectious changes in the MA site and surrounding area (mainly caused by leakage and secondary infection) can be revealed by imaging if local leakage is increased and the patient displays obvious systemic symptoms. These changes manifest as signs of air in cystic and solid lesions as well as intestinal contents and pus in the puncture fluid or drainage fluid. Such patients have a lower chance of developing bacteremia and sepsis compared with those with direct leakage into the abdominal cavity (patient numbers 2 and 8). A fistula between the intestine and urinary system is considered to be an internal IF. Due to mild symptom of fever and absence of abdominal signs and peritoneal irritation, identification of an IF by imaging is often delayed, which makes an early diagnosis quite difficult. This situation is more common following MA of a left-kidney tumor. The initial symptom is mostly mild abdominal distension, and the specific symptom is intermittent air release during urination. A diagnosis can be made if a stool is visible in urine (patient numbers 3 and 4).

### Principles for the Management and Treatment of IFs

General management includes abstinence from food and water, gastrointestinal decompression, fluid replacement and nutritional support, anti-inflammatory therapy, and surgical consultation (17, 19). Surgery must be initiated as soon as possible. For a jejunoileal injury, if inflammation at the injury site is mild, the injury site can be excised and primary anastomosis undertaken. For a colonic injury, because the blood supply to the colon is not as rich as that to the small intestine, primary healing is affected by edema and inflammation of the intestinal wall, so the risk of IF recurrence is high. In this case, one enterostomy can done first, then repositioning can be carried out if the patient is stable and inflammation subsides. For an IF formed between the intestines, MA area, and urinary system, catheter drainage can be undertaken.

### Assessment of Risk Factors for IFs

First, relevant imaging (CT/MRI) before MA must be done and the imaging results interpreted carefully. Full evaluation of the relationship between the lesion and intestines on transverse, coronal, and sagittal planes must be carried out. Full evaluation of the relationship and distance between the lesion and the intestines using image fusion and three-dimensional reconstruction techniques may be necessary.

Second, a detailed medical history and treatment history must be obtained. Adhesions and relative spatial changes in abdominal and pelvic organs can be caused by surgery in abdominal and pelvic areas, radiotherapy of the lesion and surrounding areas, peritonitis, abdominal infection, and endometrial displacement. If the lesion is in close proximity to the gastrointestinal tract and the patient has one of the medical histories mentioned above, a post-MA IF may occur due to the inability of artificial ascites to separate the adhesion in some locations.

### Prophylactic and Therapeutic Actions for IFs

If the lesion is close to the intestines and the distance between the MA site and intestines is <5 mm, auxiliary measurements should be taken to reduce the risk of intestinal injury before and during MA. Eight parameters should be considered.

#### Intestinal Cleansing

An appropriate amount of oral laxatives (e.g., polyethylene glycol electrolyte powder) should be taken after the evening mean 1 day before MA and fasting should be maintained from the morning of MA. The purposes of intestinal cleansing are to: change the relative distance between the intestines and lesion; reduce the leakage of intestinal contents after development of IF; reduce the severity of post-IF infection. The cleanliness of the intestines is beneficial for surgery.

#### Artificial Ascites

Artificial ascites can change the relative distance between the lesion and intestines, create a safe path for needle insertion, and reduce the temperature of the intestine near the MA site (4, 18, 20–22). Application of artificial ascites reduces the risk of intestinal injury greatly and ensures the feasibility and safety of MA. However, the adhesions caused by surgery, inflammation, radiotherapy, or tumor invasion may not be separated by artificial ascites, and MA may, therefore, increase the risk of IF development due to intestinal injury.

#### Path of Needle Insertion and Intestines

The relationship between the needle-insertion path and intestines should be evaluated fully before MA, especially if the small intestine lies on the needle-insertion path. Hence, the probe should be pressurized and relaxed repeatedly several times, a high-frequency probe should be adopted, or fusion images of CT/MRI and ultrasound should be used to ensure that the intestine is not on the needle-insertion path.

#### Selection of the Needle Path

The microwave electrode should not be placed with its tip pointing directly in the direction of the intestines. This strategy is used to prevent the intestinal injury caused by forward displacement of the microwave electrode due to poor visibility of the needle tip as a result of shielding by strong echoes during MA.

#### Distance Between the MA Site and Intestines

The distance between the MA site and intestines should be >5 mm. If the distance is <5 mm, temperature measurement should be undertaken concurrently during MA to ensure a temperature of <54°C near the intestines (22).

#### Other Treatments

If use of artificial ascites results in poor separation or failure to separate adhesions, other treatments should be carried out instead of MA. Alternative treatments include alcohol-injection coagulation, implantation of ^125^I particles, radiotherapy, and laparoscopic ablation can be undertaken.

#### Nutrition

After MA of lesions close to the intestines, the duration of food/water abstinence should be extended appropriately (≥24 h) (6). Multiple routine stool tests should be carried out to monitor fecal occult blood.

#### Intestinal Injury

Intestinal injury due to MA of renal and adrenal-gland lesions often lacks early specific symptoms and peritoneal irritation, and the initial symptom is often non-specific abdominal distension. Therefore, such patients should be monitored closely more than 1 month.

## Conclusions

IF often occurs following MA of lesions close to the intestines. IF is a rare complication of MA. Although the prevalence of IF is low, its consequences are often serious. A distance <5 mm between the margin of the target lesion and intestines and a history of surgery, radiotherapy of the proposed MA site and surrounding area, cirrhosis, ascites, abdominal infection, and dysmenorrhea are high-risk factors for post-MA IF.

The prevalence of IF can be reduced by undertaking three actions: (i) full evaluation of the relationship between the lesion and intestines by imaging, planning the needle-insertion path, and cleansing the intestines before MA; (ii) applying artificial ascites and measuring the real-time temperature during MA; (iii) treating tumors near the intestines with alcohol-injection coagulation, implantation of ^125^I particles, radiotherapy or by laparoscopic ablation. Surgical treatment is often required after IF development. Care should be taken if MA is to be done in patients intolerant to surgery and have a high-risk of intestinal injury following MA.

## Data Availability Statement

The original contributions presented in the study are included in the article/supplementary material, further inquiries can be directed to the corresponding author.

## Ethics Statement

The studies involving human participants were reviewed and approved by this was a retrospective study approved by the Ethics Committee of our hospital. The patients/participants provided their written informed consent to participate in this study.

## Author Contributions

ST, ZC, and XY made substantial contributions to the research design, analysis and interpretation of data. PL, ZH, YG, JZ, FL, and ZC contributed lot of the data analysis and appropriate method to the research. YG and RL took part in the building of the clinical database. ST drafted the manuscript. All the authors read and approved the final manuscript.

## Funding

Our study was supported by Grants [81871374, 82171941] from the National Scientific Foundation Committee of China, and the National Clinical Research Center for Geriatric Diseases, Chinese PLA General Hospital [NCRCG-PLAGH-2019011].

## Conflict of Interest

The authors declare that the research was conducted in the absence of any commercial or financial relationships that could be construed as a potential conflict of interest.

## Publisher's Note

All claims expressed in this article are solely those of the authors and do not necessarily represent those of their affiliated organizations, or those of the publisher, the editors and the reviewers. Any product that may be evaluated in this article, or claim that may be made by its manufacturer, is not guaranteed or endorsed by the publisher.

## References

[1] Liang P Yu J Lu MD Dong BW Yu XL Zhou XD. Practice guidelines for ultrasound-guided percutaneous microwave ablation for hepatic malignancy. World J Gastroenterol. (2013) 19:5430–8. 10.3748/wjg.v19.i33.5430 24023485 PMC3761095

[2] Liang P Wang Y Zhang D Yu X Gao Y Ni X. Ultrasound guided percutaneous microwave ablation for small renal cancer: initial experience. J Urol. (2008) 180:844–8; discussion 848. 10.1016/j.juro.2008.05.012 18635230

[3] Wang Y Liang P Yu X Cheng Z Yu J Dong J. Ultrasound-guided percutaneous microwave ablation of adrenal metastasis: preliminary results. Int J Hyperthermia. (2009) 25:455–61. 10.1080/02656730903066608 19925324

[4] Hai N Zhang J Xu R Han ZY Liu FY. Percutaneous microwave ablation with artificial ascites for symptomatic uterine adenomyosis: initial experience. Int J Hyperthermia. (2017) 33:646–52. 10.1080/02656736.2017.1285444 28118773

[5] Yang Y Zhang J Han ZY Yu MA Ma X Zhou HY. Ultrasound-guided percutaneous microwave ablation for submucosal uterine fibroids. J Minim Invasive Gynecol. (2014) 21:436–41. 10.1016/j.jmig.2013.11.012 24316137

[6] Liang P Wang Y Yu X Dong B. Malignant liver tumors: treatment with percutaneous microwave ablation–complications among cohort of 1136 patients. Radiology. (2009) 251:933–40. 10.1148/radiol.2513081740 19304921

[7] Mukthinuthalapati VP Attar B Mutneja H Syed M Gandhi S. Duodenorenal fistula after microwave ablation presenting as melena. ACG Case Rep J. (2018) 5:e76. 10.14309/02075970-201805000-00076 30426034 PMC6202425

[8] Dong X Li X Yu J Yu MA Yu X Liang P. Complications of ultrasound-guided percutaneous microwave ablation of renal cell carcinoma. Onco Targets Ther. (2016) 9:5903–9. 10.2147/OTT.S109783 27713644 PMC5045230

[9] Gao Y Liang P Yu X Yu J Cheng Z Han Z. Microwave treatment of renal cell carcinoma adjacent to renal sinus. Eur J Radiol. (2016) 85:2083–9. 10.1016/j.ejrad.2016.09.018 27776662

[10] Iliodromiti S Murage A. Multiple bowel perforations requiring extensive bowel resection and hysterectomy after microwave endometrial ablation. J Minim Invasive Gynecol. (2011) 18:118–20. 10.1016/j.jmig.2010.07.016 21195964

[11] Jamieson R Hammond I Maouris P. Small bowel perforation associated with microwave endometrial ablation. Aust N Z J Obstet Gynaecol. (2002) 42:407–8. 10.1111/j.0004-8666.2002.409_1.x 12403291

[12] Gurtcheff SE Sharp HT. Complications associated with global endometrial ablation: the utility of the MAUDE database. Obstet Gynecol. (2003) 102:1278–82. 10.1016/j.obstetgynecol.2003.07.007 14662215

[13] Brown J Blank K. Minimally invasive endometrial ablation device complications and use outside of the manufacturers' instructions. Obstet Gynecol. (2012) 120:865–70. 10.1097/AOG.0b013e31826af4fe 22996104

[14] Della Badia C Nyirjesy P Atogho A. Endometrial ablation devices: review of a manufacturer and user facility device experience database. J Minim Invasive Gynecol. (2007) 14:436–41. 10.1016/j.jmig.2007.05.008 17630160

[15] Livraghi T Meloni F Solbiati L Zanus G. Complications of microwave ablation for liver tumors: results of a multicenter study. Cardiovasc Intervent Radiol. (2012) 35:868–74. 10.1007/s00270-011-0241-8 21833809

[16] Kim KR Thomas S. Complications of image-guided thermal ablation of liver and kidney neoplasms. Semin Intervent Radiol. (2014) 31:138–48. 10.1055/s-0034-1373789 25049443 PMC4078149

[17] Wang C-C Kao J-H. Artificial ascites is feasible and effective for difficult-to-ablate hepatocellular carcinoma. Hepatol Int. (2015) 9:514–9. 10.1007/s12072-015-9639-8 26108302

[18] Akahane M Koga H Kato N Yamada H Uozumi K Tateishi R. Complications of percutaneous radiofrequency ablation for hepato-cellular carcinoma: imaging spectrum and management. Radiographics. (2005) 25(Suppl. 1):S57–68. 10.1148/rg.25si055505 16227497

[19] Zhang M Liang P Cheng ZG Yu XL Han ZY Yu J. Efficacy and safety of artificial ascites in assisting percutaneous microwave ablation of hepatic tumours adjacent to the gastrointestinal tract. Int J Hyperthermia. (2014) 30:134–41. 10.3109/02656736.2014.891765 24571176

[20] Cheng Z Yu X Han Z Liu F Yu J Liang P. Ultrasound-guided hydrodissection for assisting percutaneous microwave ablation of renal cell carcinomas adjacent to intestinal tracts: a preliminary clinical study. Int J Hyperthermia. (2018) 34:315–20. 10.1080/02656736.2017.1338362 28641464

[21] Ren C Liang P Yu XL Cheng ZG Han ZY Yu J. Percutaneous microwave ablation of adrenal tumours under ultrasound guidance in 33 patients with 35 tumours: a single-centre experience. Int J Hyperthermia. (2016) 32:517–23. 10.3109/02656736.2016.1164905 27145838

[22] Dou JP Yu J Han ZY Liu FY Cheng ZG Liang P. Microwave ablation for hepatocellular carcinoma associated with Budd-Chiari syndrome after transarterial chemoembolization: an analysis of ten cases. Abdom Radiol. (2017) 42:962–8. 10.1007/s00261-016-0923-4 27688061

